# Tumor signatures of *PTHLH* overexpression, high serum calcium, and poor prognosis were observed exclusively in clear cell but not non clear cell renal carcinomas

**DOI:** 10.1002/cam4.270

**Published:** 2014-05-26

**Authors:** Masahiro Yao, Takayuki Murakami, Koichi Shioi, Nobuhiko Mizuno, Hiroki Ito, Keiichi Kondo, Hisashi Hasumi, Futoshi Sano, Kazuhide Makiyama, Noboru Nakaigawa, Takeshi Kishida, Yoji Nagashima, Shoji Yamanaka, Yoshinobu Kubota

**Affiliations:** 1 Department of Urology and Molecular Genetics, Yokohama City University Graduate School of MedicineYokohama, Japan; 2 Department of Urology, Yokohama City University Center HospitalYokohama, Japan; 3 Department of Urology, Kanagawa Cancer CenterYokohama, Japan; 4 Department of Molecular Pathology, Yokohama City University Graduate School of MedicineYokohama, Japan; 5 Department of Surgical Pathology, Yokohama City University HospitalYokohama, Japan

**Keywords:** Gene expression, prognosis, PTHLH, qRT-PCR, renal cell carcinoma, serum calcium

## Abstract

High serum calcium (Ca) due to aberrant secretion of tumor parathyroid hormone-like hormone (PTHLH) is a well-known paraneoplastic sign and is associated with poor prognosis in patients with renal cell carcinoma (RCC). However, the status of serum Ca and tumor *PTHLH* expression have not been verified using the 2004 World Health Organization (WHO) renal tumor classification. We retrospectively reviewed corrected serum Ca levels at initial onset (*n = *683) and/or as of recurrence (*n = *71) in patients with RCC. We also examined a total of 623 renal parenchymal tumor samples for *PTHLH* mRNA expressions by quantitative real-time PCR. High serum Ca concomitant with *PTHLH* overexpression in tumors was observed exclusively in clear cell RCC but not in other non clear cell subtype tumors, including papillary, chromophobe, collecting-duct, unclassified, and other rare subtype RCCs or in benign oncocytomas and angiomyolipomas. In clear cell RCC, *PTHLH* expression was significantly high in male patients, and was associated with a symptomatic presentation, higher grade, and higher stage cases, whereas it was not associated with *VHL* gene status. Univariate analyses demonstrated that high *PTHLH* expression was strongly associated with poor outcome both in overall survival (OS) and disease-free survival (DFS) for patients who underwent standard nephrectomy. Further multivariate Cox analyses revealed that the *PTHLH* expressions remained as independent prognostic parameters for OS but not for DFS. These data suggest that the previously characterized tumor signatures of high serum Ca due to high *PTHLH* expression and poor prognosis are clear cell RCC-specific features, whereas these characteristics are rare in non clear cell RCCs.

## Introduction

Renal cell carcinoma (RCC) is the most common malignancy arising in the adult kidney 1,2. The incidence and mortality of RCC are increasing, with more than 200,000 new occurrences and 100,000 cancer deaths due to RCC estimated annually throughout the world 3.

Hypercalcemia is one of the well-known paraneoplastic manifestations in patients with RCC 2,4–6. High serum calcium (Ca) is observed in 3–17% of all RCC patients and is more frequent in the advanced and metastatic disease stages 7–9. High serum Ca has shown strong associations with tumor aggressiveness and poor prognosis; therefore, it is now widely used as one of the standardized prognostic markers for advanced and metastatic RCC patients, such as in the Memorial Sloan-Kettering Cancer Center (MSKCC) risk criteria, the National Comprehensive Cancer Network (NCCN) short survival predictors, and the more recently proposed prognostic criteria for patients treated with antiangiogenic agents 10–13.

Previous studies have demonstrated that hypercalcemia in RCC is mainly induced by the aberrant secretion of parathyroid hormone-like hormone (PTHLH) from cancer cells 14. Moreover, PTHLH has been characterized as the essential growth factor exhibiting pleiotropic effects, including tumor cell growth, differentiation, invasion, and death 15,16. Interestingly, the messenger RNA stability of the *PTHLH* gene was shown to be negatively regulated by the von Hippel-Lindau (VHL)/hypoxia-inducible factor (HIF) system via the RNA-binding protein HuR in RCC cells 16,17. The disruption of VHL that frequently occurs in clear cell RCC is therefore presumed to play an important role in the upregulation of *PTHLH* 16–18.

Data accumulated over the past 10 years have revealed that RCC consists of a number of diverse tumor subtypes. The clear cell subtype, which has the most common histology and accounts for 75–80% of all RCCs, is characterized by loss of the VHL tumor suppressor and subsequent activation of the HIF signaling pathway 1,2,18. On the other hand, tumor-associated genes other than *VHL*, such as *MET*, *FH*, *TSC1*, *TSC2*, *SDH*, and *FLCN*, are involved in other non clear cell subtype RCCs, renal oncocytoma, and angiomyolipoma (AML) 19. Due to the differences in causative genes, each renal tumor subtype presents unique morphological, biological, and clinical features. Based on these findings, a new renal tumor histologic classification was established and introduced 1,20. A precise understanding of the characteristics of each tumor subtype is becoming more and more important in the current, molecular-targeted individualized medicine 19. Although the serum Ca value is a widely used clinical biomarker for RCC, its actual status together with the tumor expression of *PTHLH* have not been well verified for each histologic subtype. In this study, we retrospectively explored these characteristics in various renal parenchymal tumors according to the 2004 World Health Organization (WHO) tumor classification 1. We also investigated the relationships between the *PTHLH* expression in tumors and, respectively, various clinicopathologic parameters, *VHL* gene alteration status, and the survival in patients with clear cell RCC.

## Patients and Methods

### Corrected serum calcium levels in patients with primary renal tumors

We retrospectively collected serum calcium (Ca) and albumin levels at initial onset and/or as of tumor recurrence just before systematic treatment in renal tumor patients consecutively treated in our hospital between July 1991 and July 2013 from the medical records. We also referred to all data on corrected serum Ca levels that were measured during the treatment course in cases with metastatic non clear cell RCC. The corrected serum Ca value was calculated using the formula proposed by Orrell 10,21. High serum Ca was defined as a corrected Ca of more than 10 mg/dL 10. The tumors were classified according to the 2004 World Health Organization Histological Classification 1. Tumor stage and grade were determined according to the 2002 Union for International Cancer Control (UICC)/TNM system, 6th edition and the Fuhrman grading system. Three patients having other apparent causes of high serum Ca, that is, one case each of chronic renal failure, multiple urolithiasis history, and functional parathyroid adenoma, were excluded from the analysis. The study protocol was approved by the institutional review board.

### Renal tumor *PTHLH* expression profiles in silico DNA microarray analyses

We searched the Gene Expression Omnibus (GEO) databases (http://www.ncbi.nlm.nih.gov/geo/) for the *PTHLH* expression levels in various renal tumors. We referred to two GEO datasets, GSE11151, and GSE15641, because both datasets contain a considerable number of renal tumors with various histologic subtypes (*n* > 60) as well as normal kidney tissue samples.

### Measurement of tumor *PTHLH* mRNA expression by real-time quantitative reverse-transcription PCR (qRT-PCR)

Primary sporadic renal tumor samples were collected from patients who had undergone standard nephrectomy at our university hospital or its affiliated hospitals under the conditions of informed consent and tissue sample availability. Fresh tumor specimens without apparent necrosis and corresponding normal kidney tissue specimens were grossly cut out immediately after nephrectomy, snap-frozen with liquid nitrogen, and stored at −80°C until nucleic acid extraction.

The isolation of total RNA from snap-frozen archived samples, preparation of cDNA, and qRT-PCR assay with a TaqMan fluorescent probe for the measurement of *PTHLH* mRNA expression levels were performed essentially as described previously 22,23. The following primers and probes spanning exons 5 to 6, which universally detected all three mRNA isoforms of the PTHLH gene, were used: 5′-GCTCGGTGGAGGGTCTCA-3′ (forward primer), 5′- TCATGGAGGAGCTGATGTTCAG-3′ (reverse primer), and 5′-6FAM- CCGCCGCCTCAAAAGAGCTGTG-Dark Quencher-3′ (probe) 14. At least two independent PCR reactions were performed to obtain the mean value. The obtained signal values were normalized by dividing them by the mean expression signal of an endogenous control gene, *β*-actin. The normalized gene expression values detected by qRT-PCR were log-transformed (on a base-2 scale) and subsequently applied to the analyses. If no *PTHLH* expression signal could be detected by the qRT-PCR, we subjected half of the signal value of the minimally expressed tumor to log-transformed data analysis.

### *VHL* gene mutation and hypermethylation in clear cell RCCs

We have previously examined renal tumors for somatic *VHL* gene alterations, including intragenic somatic mutations and promoter hypermethylations, in a series of studies 24–26. Among the clear cell RCCs examined for *PTHLH* expression in this study, *VHL* alterations were found in 132 tumors (120 intragenic mutations and 12 promoter hypermethylations) but not in 112 tumors. The detailed *VHL* mutational analyses were described in the previous reports 24–26.

### Statistical analysis

The significance of the differences between groups was assessed using Fisher's exact test, the Mann–Whitney *U* test or the Kruskal–Wallis *H* test depending on the dataset. The correlations between two parameters were tested using the Spearman's rank correlation test.

For testing the survival significance of the tumor *PTHLH* expression, a total of 542 patients with clear cell RCC who underwent standard nephrectomy were enrolled. Curative and cytoreductive nephrectomies were done in 429 and 113 patients, respectively. Clinicopathologic data, including the 2002 TNM stages, Fuhrman grade, RCC-related symptoms, age, and sex were also collected from the medical records.

Among patients with residual metastasis after cytoreductive nephrectomy (*n* = 113) or with recurrence after curative surgery (*n* = 96), additional metastasectomy was done in 54 patients. As systematic treatments, cytokine-based immunotherapies, including interferon-alpha and/or IL-2, were mainly used (*n* = 131). Molecular targeting agents, including tyrosine kinase inhibitors (TKIs) and mTOR inhibitors, were also administered in a total of 23 patients. Survival time was defined as the time from nephrectomy until the patient's death, known recurrence, or the last time that the patient was known to be alive. The median follow-up period of all patients was 70.7 months (range = 0.5–264.0 months) after nephrectomy. Survival time was estimated by the Kaplan–Meier method, and the resulting curves were assessed with the log-rank test. The Cox proportional hazards model was used to examine the simultaneous effects of several variables on patient outcome. All statistical analyses were performed using SPSS software (SPSS, Chicago, IL). All statistical tests were two-sided.

## Results

### High serum calcium in a subset of clear cell RCC patients

**Figure 1:**
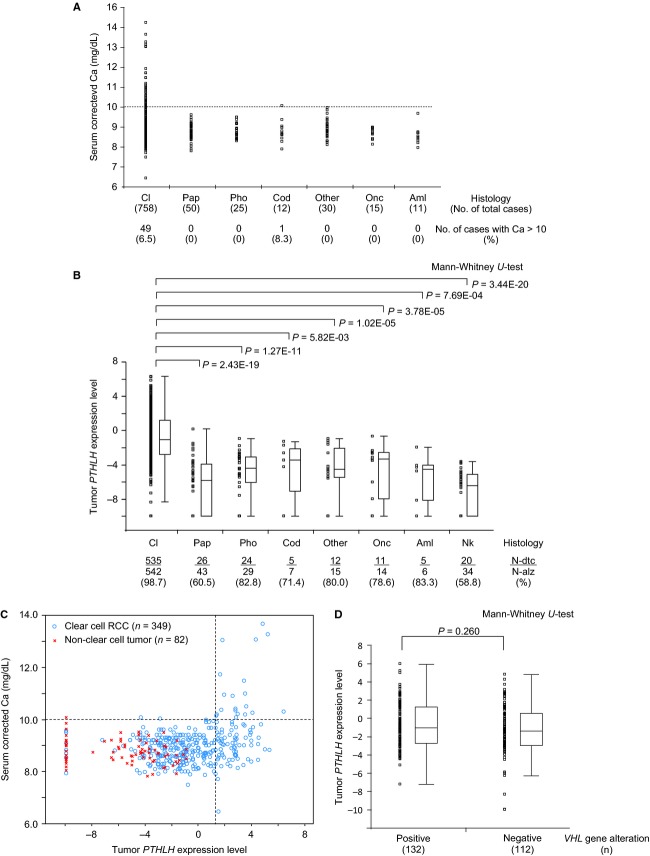
(A) Corrected serum calcium values according to renal tumor histologic subtypes. Corrected serum calcium (Ca) levels in patients with renal tumor at initial onset and/or as of the tumor recurrence were evaluated. (B) Expression levels of *PTHLH* determined by real-time quantitative PCR according to the renal tumor histologic subtypes and normal kidney tissue samples. The *PTHLH* expressions in each category are shown in a dot plot as well as a box-whisker plot. The box depicts the borders of the 25% and 75% quartiles; the horizontal bar corresponds to the median value. Whiskers represent ranges. (C) Scatter plot of the corrected serum Ca levels and tumor expression of *PTHLH* as detected by qRT-PCR in patients with clear cell RCC and non clear cell tumors. Break lines: horizontal, at 10 mg/dL of corrected serum Ca; vertical, at a *PTHLH* signal value of 1.275 in qRT-PCR. (D) *PTHLH* expression levels according to the *VHL* gene alteration status in clear cell RCCs (*n* = 246). The *VHL* gene alterations included somatic intragenic mutation and promoter hypermethylation. The *PTHLH* expressions in tumors are shown in a dot plot as well as a box-whisker plot. Cl, clear cell RCC, Pap, papillary RCC; Pho, chromophobe RCC; Cod, collecting-duct carcinoma; Other, unclassified or other rare subtype RCCs; Onc, oncocytoma; Aml, angiomyolipoma; Nk, Normal kidney tissue, N-dtc, no. of samples with detectable expression; N-alz, no. of samples analyzed.

We initially reviewed corrected serum calcium (Ca) levels in patients who presented with renal tumor at initial diagnosis and/or as of the tumor recurrence prior to systematic treatment. High serum Ca (corrected Ca > 10 mg/dL) was observed at 49/758 (6.5%) data points in patients with clear cell RCC (Fig.1A and Table1). The prevalence of high serum Ca was increased in advanced-stage and metastatic-stage (stage IV or recurrent) (Kruskal–Wallis *H* test: *P *=* *1.44E-15) or high-grade (G3 and G4) (*P *=* *5.22E-15) tumor cases in clear cell RCC, in agreement with the well-established characterization (Table1). In contrast, high serum Ca was rarely observed in the non clear cell tumor cases (clear cell vs. non clear cell tumors; Fisher's exact test: *P *=* *2.39E-03). Among the 143 Ca data points collected in non clear cell tumors—including 50 papillary, 25 chromophobe, 12 collecting-duct, 30 unclassified, or other rare subtype RCCs, 15 oncocytoma, and 11 AML cases—only one patient with recurrent collecting-duct carcinoma (#YUC667), who suffered from multiple lymph node and osteolytic bone metastases, presented with slightly high serum Ca (corrected Ca: 10.08 mg/dL) (Fig.1A and Table1). We therefore further checked the serum Ca data at up to 69 different data points during the treatment course in 22 metastatic non clear cell RCC cases. Again, the same collecting-duct carcinoma patient alone showed high serum Ca (corrected Ca: 10.13 mg/dL) at the terminal stage with multiple lymph node, lung, and bone metastases, whereas, in the other 21 metastatic non clear cell RCC cases, the serum Ca remained within the normal range throughout the treatment courses.

**Table 1:** Prevalence of high serum calcium cases according to stage and grade in each renal tumor histologic subtype

Category	No. of high serum calcium cases/no. of total case (%)						
	Renal tumor histologic subtype						
	Clear cell	Non clear cell tumor					
		Pap	Pho	Cod	Other	Onc	Aml
Stage							
I	3/412 (0.73)	0/29 (0)	0/15 (0)	0/2 (0)	0/9 (0)		
II	1/19 (5.3)	0/1 (0)	0/5 (0)	**–**	0/1 (0)		
III	3/89 (3.4)	0/8 (0)	0/4 (0)	0/1 (0)	0/4 (0)		
IV	32/164 (20)	0/7 (0)	0/1 (0)	0/4 (0)	0/3 (0)		
Rec	10/68 (15)	0/5 (0)	**–**	1/4 (25)	0/13 (0)		
NA	0/3 (0)						
Grade							
1	1/159 (0.63)	0/4 (0)	0/1 (0)	**–**	0/2 (0)		
2	9/390 (2.3)	0/29 (0)	0/18 (0)	0/1 (0)	0/8 (0)		
3	22/141 (16)	0/16 (0)	0/4 (0)	0/9 (0)	0/10 (0)		
4	13/56 (23)	**–**	0/2 (0)	1/2 (50)	0/2 (0)		
NA	4/12 (33)	0/1 (0)			0/8 (0)		
All data point	49/758 (6.5)	0/50 (0)	0/25 (0)	1/12 (8.3)	0/30 (0)	0/15 (0)	0/11 (0)

Stage, 2002 UICC staging system; Grade, Fuhrman grade; Rec, recurrence; NA, not available; Pap, papillary; Pho, chromophobe; Cod, collecting-duct; Other, unclassified or other rare subtype RCC; Onc, oncocytoma; Aml, angiomyolipoma.

### Upregulation of the *PTHLH* gene in clear cell RCCs

It has been well characterized that the main cause of hypercalcemia in RCC is the aberrant secretion of PTHLH from tumor cells 4–6. Therefore, we referred to the *PTHLH* expression profiles of renal tumor in the previously published DNA microarray datasets (the GEO datasets, GSE11151, and GSE15641; Table S1). In these two independent datasets, markedly high *PTHLH* signals were observed in some clear cell RCCs, whereas, in the other histologic subtype tumors as well as in the normal kidney tissue samples, the *PTHLH* signals appeared to be low.

To verify these preliminary observations from the microarray datasets, we measured *PTHLH* mRNA expression levels in various renal parenchymal tumors by quantitative real-time PCR (qRT-PCR). A total of 656 primary renal tumors—that is, 542 clear cell, 43 papillary, 29 chromophobe, seven collecting-duct, 15 unclassified, or other rare subtype RCCs, 14 oncocytomas, and 6 AMLs—as well as 34 normal kidney tissue samples were analyzed for *PTHLH* mRNA expression. The qRT-PCR assay demonstrated that, among renal tumor subtypes, the clear cell RCCs presented with a higher incidence of detectable signals of *PTHLH* (535/542 tumors; 98.7%) compared to the other tumor subtypes (60.5–83.3%) as well as to normal kidney samples (58.8%) (Fig.1B). As for the expression signal levels, the clear cell RCCs again presented with significantly higher values compared to the other renal tumor subtypes and normal kidney tissues (Fig.1B).

### High serum Ca concomitant with high *PTHLH* expression in clear cell but not in non clear cell subtype renal tumors

We next analyzed the possible correlation between the levels of corrected serum Ca and tumor expression of *PTHLH*. Among our tumor cases, the serum Ca data concomitant with tumor *PTHLH* expression were measured in 349 clear cell and 82 non clear cell tumors. The correlation studies demonstrated a weak association between serum Ca and *PTHLH* expression levels in the clear cell RCC cases (Spearman's rank correlation coefficient: *ρ *= 0.192, *P = *3.19E-04), but not in non clear cell tumors (*ρ *= −0.225, *P = *0.0422) (Fig.1C). It is notable that, in clear cell RCCs, 20/22 (90.9%) patients presenting with high serum Ca suffered from the high *PTHLH* expression in tumors, that is, a *PTHLH* signal value >1.275 in the qRT-PCR assay (Fig.1C). In contrast, all non clear cell tumor cases were plotted in the normocalcemia range with a relatively low-expression of *PTHLH* (≤0.213) except for one collecting-duct carcinoma case (#YUC667) (Fig.1C). Although, as mentioned previously, this patient presented clinically with slightly high serum Ca, the qRT-PCR assay revealed that the tumor expression of *PTHLH* was at an undetectable level.

### Correlation study of tumor *PTHLH* expression and clinicopathologic characteristics and *VHL* gene alteration in clear cell RCCs

We next explored possible correlations between the level of *PTHLH* expression in tumors and various clinicopathologic characteristics and *VHL* gene alteration status in clear cell RCC cases. The *PTHLH* expression in tumors was significantly higher in male patients and in case with positive symptoms, higher stage tumors, and higher grade tumors (Table2).

**Table 2:** Correlations between clinicopathologic characteristics and *VHL* gene alteration and tumor PTHLH expression in patients with clear cell RCC

Characteristic (*n*)	*n* (%)	*PTHLH* expression in qRT-PCR		*P*
		Median	Interquartile range	
Age, year (542)				
<65	317 (59)	−0.029	4.119	0.295^1^
65≤	225 (41)	−0.620	4.281	
Sex (542)				
Female	149 (27)	−1.442	3.778	0.0470^1^
Male	393 (73)	−0.967	4.039	
RCC-related symptom (542)				
Asymptomatic	324 (60)	−1.280	3.470	4.33E-03^2^
Local	142 (26)	−0.589	3.863	
Systematic	76 (14)	−0.140	4.875	
2002 UICC stage (542)				
I	299 (55)	−1.379	3.524	9.34E-06^2^
II	22 (4.1)	−1.104	3.067	
III	92 (17)	−0.835	3.802	
IV	129 (24)	0.336	3.990	
Fuhrman grade (542)				
1	116 (21)	−1.324	3.359	7.51E-05^2^
2	276 (51)	−1.273	3.495	
3	112 (21)	0.273	3.953	
4	38 (7.0)	−0.491	5.442	
*VHL* gene alteration (244)				
Positive	132 (54)	−1.046	3.990	0.260^1^
Negative	112 (46)	−1.402	3.493	

UICC, Union for International Cancer Control.

1 Mann–Whitney *U* test.

1 Kruskal–Wallis *H* test.

Previous studies have demonstrated that the *PTHLH* mRNA is up-regulated by inactivation of the *VHL* gene 16,17. We therefore examined the association between the *VHL* gene alteration status (intragenic mutation and promoter hypermethylation) and the tumor expression of *PTHLH* in selected clear cell RCCs (*n* = 244). However, *PTHLH* expression did not differ significantly between the negative and positive *VHL* alteration tumor groups (Table2). Of note, marked variations of *PTHLH* expression signals, from very high to almost undetectable levels, were observed even in the *VHL* alteration-positive tumor group (Fig.1D).

### Correlation of high *PTHLH* expression and poor survival in clear cell RCCs

**Figure 2:**
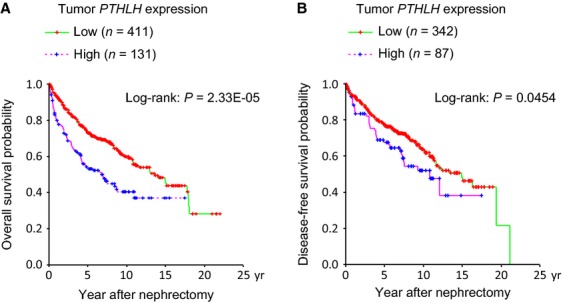
Kaplan–Meier plots of estimated survivals and tumor expression of *PTHLH* in patients with clear cell RCC who underwent nephrectomy. (A) Overall survivals for all patients (*n* = 542), and (B) disease-free survival of patients who underwent presumably curative surgery (*n* = 429). Low, *PTHLH* expression value in qRT-PCR = <1.275; High, *PTHLH* expression value in qRT-PCR > 1.275.

Finally, we examined whether the tumor expression of *PTHLH* was correlated with the survival of patients with clear cell RCC, who underwent standard nephrectomy. We defined the cutoff value of the tumor *PTHLH* expression signal detected by qRT-PCR as 1.275 because of the finding that the majority of the clear cell RCC cases with high serum Ca belonged above this signal value in the correlation scattergram (Fig.1C). Tumors with PTHLH expression signal value of >1.275 were therefore considered to be a high-expression group, while those with signal value of ≤1.275 were considered to be a low-expression group. Kaplan–Meier analyses demonstrated that high *PTHLH* expression levels were strongly associated with poor overall survival (OS) for all patients (*n* = 542) (Log-rank test: *P = *2.33E-05; median survival period: 164.0 vs. 82.9 months) and disease-free survival (DFS) for patients who underwent curative nephrectomy (*n* = 429) (Log-rank: *P *=* *0.0454; median survival period: 179.4 vs. 130.1 months) (Fig.2A and B). Further, Cox multivariate analyses including sex, RCC-related symptoms, stage, grade, and PTHLH, demonstrated that high *PTHLH* expression still remains a statistically significant independent parameter of worse outcome in terms of OS (*P* = 0.001) (Table3). On the other hand, the prognostic impact of PTHLH on DFS was weak and we failed to detect any statistical significance in the multivariate regression model (*P* = 0.162) (Table4).

**Table 3:** Cox univariate and multivariate analyses of overall survival among 542 patients with clear cell RCC who underwent nephrectomy

Characteristic	*n* (%)	Univariate analysis			Multivariate analysis		
		*P*	HR	95% CI	*P*	HR	95% CI
Sex							
Female	149 (28)		1.00			1.00	
Male	393 (72)	0.376	1.15	0.85–1.56	0.777	1.05	0.77–1.42
RCC-related symptom							
Asymptomatic	324 (60)		1.00			1.00	
Local	142 (26)	<0.001	3.14	2.29–4.31	0.011	1.60	1.11–2.28
Systemartic	76 (14)	<0.001	7.28	5.17–10.26	0.001	1.99	1.31–3.04
2002 UICC stage							
I	299 (55)		1.00			1.00	
II	22 (4.1)	0.802	0.89	0.35–2.23	0.516	0.73	0.29–1.88
III	92 (17)	<0.001	2.30	1.53–3.44	0.008	1.76	1.16–2.67
IV	129 (24)	<0.001	9.93	7.19–13.72	<0.001	5.09	3.42–7.58
Fuhrman grade							
1	116 (21)		1.00			1.00	
2	276 (51)	0.011	1.90	1.16–3.12	0.456	1.21	0.73–2.02
3	112 (21)	<0.001	6.66	4.02–11.05	0.003	2.28	1.31–3.97
4	38 (7.0)	<0.001	11.78	6.69–20.74	<0.001	4.25	2.31–7.82
*PTHLH* expression							
Low	411 (76)		1.00			1.00	
High	131 (24)	<0.001	1.85	1.38–2.47	0.001	1.65	1.23–2.22

**Table 4:** Cox univariate and multivariate analyses of disease-free survival among 429 patients with clear cell RCC who underwent nephrectomy

Characteristic	*n* (%)	Univariate analysis			Multivariate analysis		
		*P*	HR	95% CI	*P*	HR	95% CI
Sex							
Female	122 (28)		1.00			1.00	
Male	307 (72)	0.159	1.31	0.90–1.91	0.284	1.23	0.84–1.81
RCC-related symptom							
Asymptomatic	310 (72)		1.00			1.00	
Local	99 (23)	<0.001	2.70	1.91–3.82	<0.001	2.01	1.39–2.92
Systemartic	20 (4.7)	0.008	2.45	1.27–4.74	0.797	1.10	0.54–2.21
2002 UICC stage							
I	299 (70)		1.00			1.00	
II	22 (5.1)	0.542	1.27	0.59–2.76	0.983	1.01	0.45–2.25
III	92 (21)	<0.001	2.53	1.76–3.64	0.001	1.96	1.34–2.86
IV	16 (3.7)	<0.001	7.26	4.14–12.71	<0.001	5.53	3.00–10.18
Fuhrman grade							
1	112 (26)		1.00			1.00	
2	237 (55)	0.006	2.05	1.23–3.43	0.034	1.75	1.04–2.95
3	62 (15)	<0.001	5.80	3.29–10.24	<0.001	3.82	2.10–6.95
4	18 (4.2)	<0.001	8.06	3.99–16.26	<0.001	5.22	2.54–10.73
*PTHLH* expression							
Low	342 (80)		1.00			1.00	
High	87 (20)	0.045	1.47	1.01–2.14	0.163	1.32	0.89–1.96

## Discussion

In this study, we demonstrated that high serum Ca and high *PTHLH* expression in tumors, the major etiology of hypercalcemia in malignancy, were observed in clear cell RCCs but not in other non clear cell tumors when using the 2004 WHO renal tumor classification. We found that when cases with clear cell RCC showed a clinical manifestation of high serum Ca, the majority of tumors expressed a substantially high level of *PTHLH* mRNA—i.e., a *PTHLH* expression signal of >1.275 by our qRT-PCR assay. On the other hand, none of the non clear cell tumors expressed such a high level of the *PTHLH* gene. In clear cell RCCs, the *PTHLH* expression levels in the tumor were positively correlated with symptomatic presentation, stage and grade as well as with poor patient OS. These findings strongly suggest that the previously characterized tumor signatures of high serum Ca due to high *PTHLH* expression and poor prognosis are clear cell RCC-specific features, whereas these characteristics are extremely rare in other non clear cell RCCs.

Despite the robust evidence of a triangular relationship among high PTHLH secretion, hypercalcemia, and patient poor prognosis, the relevance of PTHLH expression to the survival of patients with RCC was not clear in the previous analyses. Iwamura et al. 27 reported that RCC patients whose tumor samples showed strong positive immunostaining for PTHLH had longer recurrence-free survivals. On the other hand, Papworth et al. 28,29 found that the serum PTHLH value was positively correlated with the serum calcium levels, but neither the serum PTHLH level nor the PTHLH tumor immunostaining level provided additional prognostic information in the multivariate regression models. In contrast, we demonstrated that high *PTHLH* expression in tumors was strongly associated with poor OS for all stages of clear cell RCC. We found that the tumor *PTHLH* expression still provided additional prognostic information when compared directly to other established parameters, including TNM stage, Fuhrman grade, and RCC-related symptoms, in the multivariate regression model. The most probable reason for the discrepancy between the previous results and our current data may be the differences among the RCC patient cohorts analyzed. That is, we focused on a patient population who suffered from histologically confirmed clear cell RCC and underwent nephrectomy as an initial treatment, whereas previous studies included patients with non clear cell RCC and/or without nephrectomy. As shown in this study, in the majority of non clear cell RCC cases, *PTHLH* expression would be expected to be low and not associated with patient survivals. Another reason may be the differences in the PTHLH detection procedures. We measured tumor *PTHLH* expression by qRT-PCR, a highly sensitive gene expression detection assay with a relatively wide dynamic range, while previous researchers used immunohistochemistry of tissue specimens or detection of serum PTHLH proteins 27–29. Our data suggest that the quantitative determination of the tumor *PTHLH* expression may reflect tumor biological characteristics—that is, aggressive and non aggressive phenotypes—more directly and would provide useful prognostic information.

Previous in vitro studies demonstrated that *PTHLH* mRNA was stabilized and up-regulated by the HuR mRNA-stabilizing protein, which is induced first by the loss of VHL and subsequently by activation of the HIF transcription factor 16,17. However, we did not find any apparent associations between *VHL* gene status and *PTHLH* expression levels in clear cell RCC. Rather, our data suggested that both the *PTHLH* upregulation and high serum Ca occurred as relatively late events during the tumor progression and were associated with aggressive phenotype. The inactivation of VHL is found in 50–80% of clear cell RCCs and is considered to be an essential, first-step molecular alteration in the tumorigenic pathway for this tumor subtype 25,30,31. Taken together, these results suggest that, in addition to *PTHLH* mRNA stabilization by dysregulation of VHL/HIF/HuR system, an additional molecular change is needed for the activation of *PTHLH* as a later event. Previous extensive analyses have demonstrated that the *PTHLH* gene is regulated by relatively complicated molecular mechanisms 14,32. The *PTHLH* gene has three distinctive promoters, that is, P1, P2, and P3, and is potentially transactivated by various transcription factors, including TATA, ETS, Sp1, cAMP-responsive element (CRE), and vitamin D receptor (VDR) 32. On the other hand, Holt et al. reported that the promoter methylation status also modulates *PTHLH* gene expression levels in RCC cells 33. Collectively, these results show that complicated genetic and epigenetic regulations underlie the activation and/or repression of *PTHLH* in a renal tumor subtype-specific manner and should be further clarified.

The biochemical marker of serum Ca as well as the risk stratifications including this parameter, such as the MSKCC risk criteria and the NCCN short survival predictors have been widely applied to predict the survival period, to choose among treatment options, and for the registry of clinical trials regardless of renal tumor histologic subtypes 34,35. However, from our present data, the application of serum Ca as a clinical marker may not be suitable for non clear cell subtypes, which make up 20–25% of all RCCs. Further studies, including analyses with a substantial number of non clear cell RCCs of advanced metastatic stage, will be needed to validate our findings. In addition, it will be of interest to evaluate both the prognostic and predictive utilities of *PTHLH* expression, as a molecular biomarker, for advanced and metastatic clear cell RCC treated with current molecular targeting agents.

## Supporting Information

**Table S1.** *PTHLH* expression profiles of renal tumor in the published DNA microarray datasets (the GEO datasets, GSE11151 and GSE15641).
